# Long-term prognostic value of quantitative myocardial perfusion in patients with chest pain and normal coronary arteries

**DOI:** 10.1007/s12350-018-1448-8

**Published:** 2018-10-04

**Authors:** Andrea G. Monroy-Gonzalez, R. A. Tio, J. C. de Groot, H. H. Boersma, N. H. Prakken, M. J. L. De Jongste, E. Alexanderson-Rosas, R. H. J. A. Slart

**Affiliations:** 1 Medical Imaging Centre, University of Groningen, University Medical Center Groningen, Hanzeplein 1, Afdeling NGMB HPC EB5, Postbus 30.001, 9700 RB Groningen, The Netherlands; 2 Department of Cardiology, Catharina Hospital Eindhoven, Eindhoven, The Netherlands; 3 Department of Cardiology, University of Groningen, University Medical Center Groningen, Groningen, The Netherlands; 4 Department of Nuclear Medicine, National Institute of Cardiology Ignacio Chavez, Mexico City, Mexico; 5 Department of Physiology, The National Autonomous University of Mexico, Mexico City, Mexico; 6 Department of Biomedical Photonic Imaging, Faculty of Science and Technology, University of Twente, Enschede, The Netherlands

**Keywords:** Microvascular dysfunction, PET, Myocardial blood flow, Diagnostic and prognostic application

## Abstract

**Background:**

Patients with chest pain and no obstructive coronary artery disease have shown a high incidence of major adverse cardiovascular events (MACE). We evaluated the role of absolute myocardial perfusion quantification in predicting all-cause mortality and MACE during long-term follow-up in this group of patients.

**Methods:**

We studied 79 patients who underwent Nitrogen-13 ammonia PET for quantification of global myocardial blood flow (MBF) and myocardial flow reserve (MFR) due to suspected impaired myocardial perfusion. Patients with coronary artery disease (i.e., > 30% stenosis in one or more coronary arteries) were excluded. We assessed all-cause mortality and MACE. MACE was defined as the composite incidence of death, myocardial infarction (MI), or hospitalization due to heart failure.

**Results:**

Median follow-up was 8 (IQR: 3-14) years. Univariate Cox regression showed that only MFR (*P* = 0.01) was a predictor of all-cause mortality. Univariate Cox regression analysis showed that both MFR and Stress MBF were predictors of the composite endpoint of MACE (*P* < 0.001 and *P* = 0.01, respectively).

**Conclusion:**

Quantitative assessment of myocardial perfusion may predict all-cause mortality and MACE in patients with chest pain and normal coronary arteries in the long-term follow-up.

**Electronic supplementary material:**

The online version of this article (10.1007/s12350-018-1448-8) contains supplementary material, which is available to authorized users.

## Spanish Abstract

**Antecedentes:**

Los pacientes con dolor torácico y sin enfermedad arterial coronaria obstructiva tienen una alta incidencia de eventos cardiovasculares adversos mayores (ECAM). Evaluamos el papel de cuantificación absoluta de perfusión miocárdica para predecir la mortalidad por cualquier causa y por ECAM en el seguimiento a largo plazo en este grupo de pacientes.

**Métodos:**

Estudiamos 79 pacientes a quienes se les realizó PET con 13-Nitrógeno Amonia para la cuantificación absoluta del flujo sanguíneo miocárdico global (FSM) y la reserva de flujo miocárdica (RFM) por sospecha de alteraciones en la perfusión miocárdica. Se excluyeron a los pacientes con enfermedad arterial coronaria (estenosis > 30% en una o más arterias). Evaluamos las causas de mortalidad y de ECAM. ECAM fue definida como la incidencia combinada de muerte, infarto al miocardio (IM) u hospitalización por falla cardíaca.

**Resultados:**

La media de seguimiento fue de 8 años (IQR: 3-14). La regresión univariada COX mostró que solo la RFM, es un predictor de mortalidad por cualquier causa. El análisis de regresión univariada Cox mostró que tanto la RFM como la FSM en estrés, fueron predictores para el desenlace de ECAM (*P* < 0.001 y *P* = 0.01, respectivamente).

**Conclusión:**

La evaluación cuantitativa de la perfusión miocárdica, puede predecir la mortalidad por cualquier causa y por ECAM en pacientes con dolor torácico y arterias coronarias normales en el seguimiento a largo plazo.

## Chinese Abstract

**背景:**

患有胸痛但无阻塞性冠状动脉疾病的患者仍存在较高的主要不良心血管事件 (MACE) 发生率。 本研究评估了心肌灌注绝对定量对该组患者长期随访期间全因死亡率和 MACE的预测价值。

**方法:**

共纳入79例疑似心肌缺血患者, 行^13^N-氨水 PET 心肌灌注显像获得定量的心肌血流值 (MBF) 和心肌血流储备 (MFR)。 排除冠状动脉疾病 (即一支或多支冠状动脉狭窄 > 30%) 的患者。 分析全因死亡率和 MACE。 MACE 定义为死亡,心肌梗死 (MI) 或因心力衰竭住院的复合事件。

**结果:**

随访中位时间为8年 (IQR:3-14)。 单变量Cox回归分析显示只有 MFR (*P* = 0.01) 是全因死亡率的预测因子。而 MFR 和负荷MBF是 MACE 复合终点事件的预测因子 (分别为 *P* < 0.001和 *P* = 0.01)。

**结论:**

在有胸痛但冠状动脉正常患者的长期随访中, 心肌灌注血流定量分析可预测全因死亡率和 MACE。

## French Abstract

**Contexte:**

A certain nombre de patients présentant des douleurs thoraciques sans évidence de coronaropathie obstructive ont une incidence élevée d’événements cardiovasculaires indésirables majeurs. Nous avons étudié le rôle de la quantification absolue de la perfusion myocardique en tant que valeur prédictive de mortalité et d’événements cardiovasculaires majeurs indésirables chez ces patients.

**Méthodes:**

Nous avons étudié 79 patients suspect de maladie coronarienne. Nous avons évalué de manière quantitative leur perfusion myocardique globale et leur réserve de débit myocardique (MFR) par tomographique à positrons à l’ammoniac marqué à l’azote 13. Les patients atteints de maladie coronarienne définie par une sténose de plus de 30% d’une ou de plusieurs artères coronaires ont été exclus. Nous ont évalué la mortalité globale (quelqu’en soient les causes) et les événements cardiovasculaires majeurs indésirables

**Résultats:**

Le suivi moyen fut de 8 ans (3-14 ans). L’analyse des données au moyen de la régression univariée de Cox a montré que seul le flux de réserve myocardique (*P* = 0.01) est un facteur prédictif de mortalité, toutes causes confondues. La même régression statistique a également permis de démontrer que la perfusion myocardique globale et la réserve de débit coronaire sont des paramètres prédictifs déterminant pour la survenue événements cardiovasculaires majeurs indésirables majeurs (*P* < 0.001 et *P* = 0.01 respectivement).

**Conclusion:**

L’évaluation quantitative de la perfusion myocardique des patients présentant des douleurs thoraciques et chez qui les artères coronaires s’avèrent normales permet de prédire le risque de mortalité et la survenue d’événements cardiovasculaires majeurs indésirables dans le suivi à long terme.

## Introduction

Many patients with angina pectoris have normal or near-normal coronary arteries. Approximately 50% of women and 30% of male patients referred to invasive coronary angiography due to suspected obstructive coronary artery disease (CAD) do not present significant coronary stenosis.^1^–^3^ It is reported that this group of patients may have a high incidence of major adverse cardiovascular events (MACE) when compared to an asymptomatic reference population.^4^,^5^ Furthermore, it is suggested that in at least half of these patients microvascular dysfunction is accountable for the symptoms, also called microvascular angina.^2^

Meanwhile, vasodilator capacity of the myocardial microvasculature can be quantified by stress myocardial blood flow (MBF) and myocardial flow reserve (MFR) measured by Positron Emission Tomography (PET).^2^,^6^ Stress MBF and MFR have shown to be reliable predictors of outcomes, independent of the presence of significant stenosis.^7^–^11^ However, whether stress MBF and MFR measurements can predict MACE in patients with chest pain and no obstructive CAD during long-term follow-up is unknown.

The aim of our study was to evaluate whether stress MBF and/or MFR are able to predict all-cause mortality and MACE at long-term follow-up in patients with chest pain and normal or near-normal coronary arteries.

## Methods

We retrospectively studied 79 consecutive patients with chest pain, history of normal or near-normal coronary arteries, and suspected impaired myocardial perfusion. All patients underwent Nitrogen-13 ammonia PET for perfusion quantification at the University Medical Center Groningen, between 1994 and 2015. Patients had a history of angina pectoris (typical or atypical) and normal or near-normal coronary arteries, as demonstrated by invasive coronary angiography and/or coronary computed tomography angiography, within 12 months before or after the Nitrogen-13 ammonia PET. Patients with CAD > 30% stenosis, left bundle branch block, severe valve heart disease, hypertrophic cardiomyopathy, and other types of cardiomyopathy with left ventricular ejection fraction < 35% were excluded. The study was conducted in accordance with the standards of the local ethics committee. For this retrospective study design, a study formal consent was not required.

### PET Imaging Acquisition

Fifty (63%) patients between 1993 and 2005 were studied in an ECAT-951/31 PET system (Siemens/CTI, Knoxville, Tennessee, USA), as previously reported.^12^ Twenty-three (29%) patients between 2005 and 2009 were studied in an ECAT Exact HR + PET camera (Siemens, Hoffman Estates, IL, USA), as previously reported.^13^ Six (8%) patients between 2009 and 2015 were studied in a whole-body 64-slice PET/CT scanner Biograph True Point (Siemens Healthcare, Erlangen, Germany), as previously reported.^14^ In brief, imaging acquisition started in resting conditions after a Nitrogen-13 ammonia i.v. injection. Following the part of the study at rest, pharmacological stress was induced with dipyridamole or adenosine. After the pharmacological stress, a second dose of Nitrogen-13 ammonia was injected i.v. and imaging acquisition was performed. Dynamic datasets were obtained at rest and under stress conditions. Dynamic rest and stress MBF data were expressed in ml/gr/min myocardial tissue. Myocardial blood flow quantifications were assessed by the Hutchins 2-tissue-compartment model.^15^ MFR was quantified as the ratio of stress MBF to rest MBF. Abnormal MFR was considered < 2.0 and abnormal stress MBF was considered < 1.9 ml/gr/min.^16^

### Follow-up

Clinical data were retrieved from electronic medical records. Patients were followed until June 2016. All-cause mortality was assessed during long-term follow-up after PET scan. Because the electronic medical records are linked to the Municipal Personal Records Database (GBA), which reports the date of death registered by the government, long-term follow-up for all-cause mortality was achieved in all patients. Cardiac death was considered as sudden death, unknown death but cardiac death not excluded, or any cause of death attributable to a cardiovascular cause. For the secondary endpoint of this study, we studied the composite incidence of MACE assessed as cardiac death, hospitalization due to heart failure, myocardial infarction (MI), and/or late revascularization (after 90 days of PET acquisition). MI was considered as only events meeting the criteria of the third universal definition of myocardial infarction.^17^ Early driven revascularization (percutaneous coronary intervention or coronary bypass grafting) within 90 days post PET acquisition was considered driven by the imaging study and excluded from the analysis.

### Statistical Analysis

Continuous variables are presented as a mean and standard deviation. Categorical variables are presented as simple proportions. Student *t* test and one-way ANOVA test were used to compare continuous variables. Chi square and Fisher tests were used to compare proportions of variables. Overall mortality and MACE were assessed using the Kaplan–Meier method. The log-rank test was used to compare survival among groups. Univariate Cox regression analysis was used to identify predictors of all-cause mortality and MACE and adjustment for possible confounders was performed when considered necessary. For the evaluation of MACE, patients were censored at the time of the first event. Multiple imputation was used in order to conduct a sensitivity analysis for missing data. A 2-tailed *P* value ≤ 0.05 was considered statistically significant. All statistical analyses were performed using SPSS v23.

## Results

Baseline characteristics of the 79 patients are summarized in Table 1. Mean rest MBF was 1.1 ± 0.3 ml/gr/min, mean stress MBF was 2.1 ± 0.6 ml/gr/min, and MFR was 2.2 ± 0.8. 34 (43%) patients had an abnormal MFR (< 2.0), and 29 (37%) patients had an abnormal stress MBF (< 1.9 ml/gr/min). Baseline characteristics were similar among patients that underwent a scan in different cameras (ECAT-951/31 PET, ECAT Exact HR + PET, and PET/CT scanner Biograph True Point); however, rest MBF, stress MBF, and MPR showed significant differences among the three cameras (Supplementary Table 1). While follow-up for all-cause mortality was achieved in all the 79 (100%) patients, complete close follow-up for MACE was only achieved in 44 (56%) patients.

**Table 1:** Baseline characteristics of patients

	All patients (*N* = 79)	Patients with normal MFR (*N* = 45)	Patients with abnormal MFR (*N* = 34)	*P* value
Age (years)	51 ± 11 years	51 ± 11 years	51 ± 11 years	0.94
Female gender	59 (74%)	32 (71%)	27 (79%)	0.40
Diabetes Mellitus	3 (4%)	2 (4%)	1 (3%)	0.73
Hypertension	27 (34%)	15 (36%)	11 (32%)	0.76
Dyslipidemia	22 (28%)	12 (27%)	10 (29%)	0.79
Smoker	14 (18%)	4 (9%)	10 (29%)	0.02
Body mass index	26 ± 5	27 ± 5	26 ± 5	0.73
Typical angina	43 (54%)	26 (60%)	17 (40%)	0.49
Dyspnea	31 (39%)	26 (54%)	22 (46%)	0.53

### All-Cause Mortality

Median follow-up time of our patients was 8 (IQR: 4-14) years. During follow-up, six (8%) out of the 79 patients died. Cardiac death occurred in four (5%) patients. Non-cardiac death occurred in two (3%) patients.

**Figure 1:**
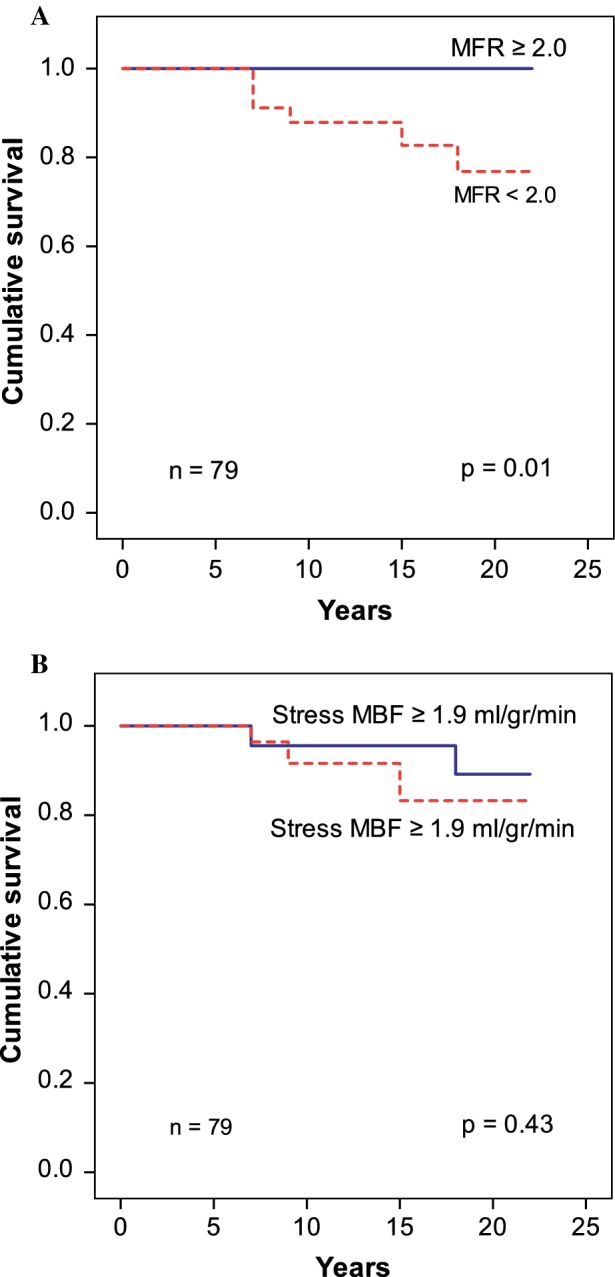
Kaplan Meier curves (*N* = 79) showing a significantly higher all-cause mortality in patients with low MFR (**A**). Meanwhile, all-cause mortality was similar in patients with normal and abnormal stress MBF (**B**)

Unadjusted Kaplan–Meier analysis demonstrated a significant increase of death events in patients with an MFR < 2.0 but not in patients with abnormal stress MBF < 1.9 ml/gr/min (*P* = 0.01 and *P* = 0.43 respectively) (Figure 1A, B, respectively). Univariate Cox regression showed that only MFR (*P* = 0.001) was a predictor of all-cause mortality (Table 2). Remaining clinical characteristics were not statistically significant predictors of all-cause mortality. DM was excluded from the Univariate Cox regression analysis because the model did not converge. The three patients with DM did not die during follow-up. Camera-adjusted analysis showed similar results, including that MFR was a predictor of MACE while stress MBF was not [HR 0.35 (0.12-1.00), *P* = 0.05, and 0.05 (0.004-0.62), *P* = 0.40]. Differences in clinical characteristics among patients with a normal and an abnormal MFR are shown in Table 1.

**Table 2:** Univariate Cox regressions showing predictors of all-cause mortality

*N* = 79	Hazard ratio	Lower 95% CI	Upper 95% CI	*P* value
Age	1.07	0.99	1.16	0.08
Female gender	1.73	0.20	14.83	0.62
Hypertension	2.14	0.43	10.68	0.35
Dyslipidemia	2.38	0.48	11.85	0.29
Smoker	1.60	0.29	9.01	0.59
Typical angina	2.75	0.50	15.04	0.24
Dyspnea	1.97	0.39	9.80	0.41
Increment per unit of stress MBF (ml/gr/min)	0.36	0.07	1.75	0.20
Increment per unit of MFR	0.05	0.01	0.30	0.001

*MBF*, myocardial blood flow; *MFR*, myocardial flow reserve

### Major Adverse Cardiac Events

**Figure 2:**
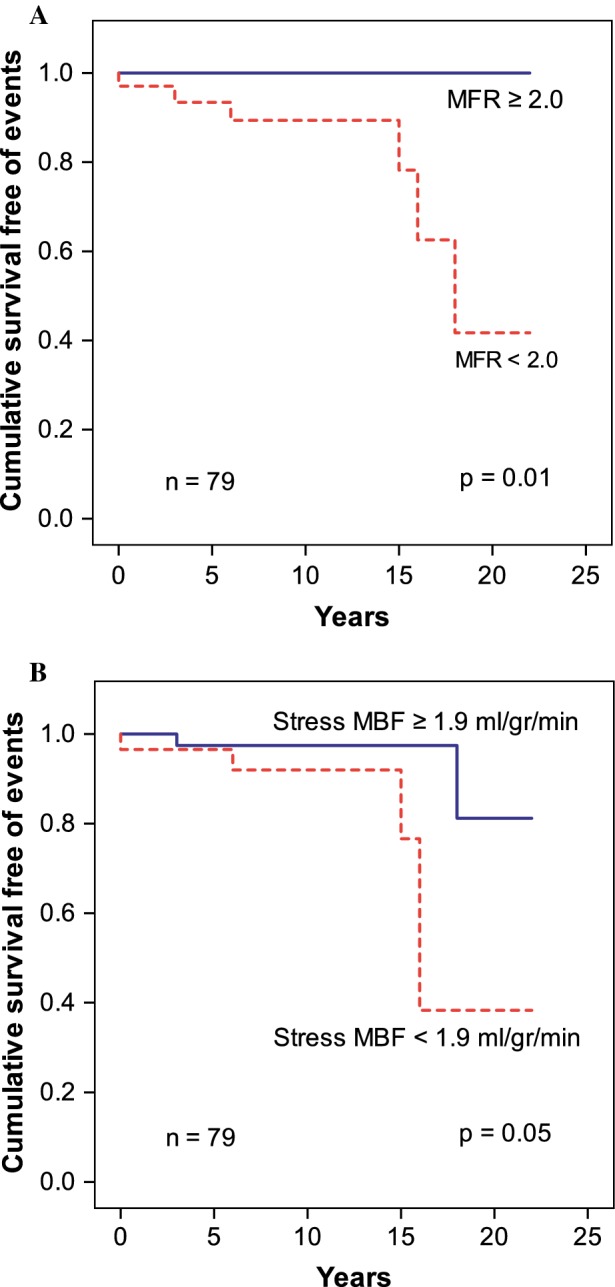
Kaplan Meier curves showing a significantly higher incidence of MACE in patients with low MFR (**A**) and low stress MBF (**B**)

MACE was reported in six (8%) out of the 79 patients. Hospitalization due to MI occurred in three (4%) patients, late revascularization was performed in two patients (3%) and cardiac death occurred in one (1%) patient. Unadjusted Kaplan–Meier analysis demonstrated a significant increase of MACE in patients with an abnormal MFR (*P* = 0.01) and abnormal stress MBF (*P* = 0.05) (Figure 2A, B, respectively). Univariate Cox regression showed that MFR and stress MBF are predictors of MACE (Table 3). DM was excluded from the Univariate Cox regression analysis because the model did not converge. The three patients with DM did not present MACE during the follow-up period. Camera-adjusted analysis showed similar results, including that MFR and stress MBF are predictors of MACE [HR 0.01 (0.001-0.33), *P* = 0.01, and 0.05 (0.005-0.67), *P* = 0.02]. Because 35 (44%) patients were lost to follow-up, different sensitivity analyses were performed demonstrating similar results (Supplementary Tables 2 and 3). Patients with hypertension were more frequent in the group with complete follow-up than in the group with incomplete follow-up [20 (46%) vs 7 (20%), *P* = 0.02]. Remaining clinical characteristics and quantitative myocardial perfusion measurements of patients with a complete follow-up were similar to patients with an incomplete follow-up (data not shown).

**Table 3:** Univariate Cox regressions showing predictors of MACE

*N* = 79	Hazard ratio	Lower 95% CI	Upper 95% CI	*P* value
Age	1.07	0.99	1.16	0.08
Female gender	1.64	0.19	14.09	0.65
Hypertension	2.43	0.47	12.42	0.29
Dyslipidemia	2.38	0.48	11.85	0.29
Smoker	1.45	0.26	8.24	0.67
Typical angina	1.35	0.27	6.73	0.71
Dyspnea	1.73	0.35	8.63	0.50
Increment per unit of stress MBF (ml/gr/min)	0.14	0.03	0.81	0.03
Increment per unit of MFR	0.03	0.004	0.28	< 0.01

*MBF*, myocardial blood flow; *MFR*, myocardial flow reserve

## Discussion

The present study shows that a decreased MFR (< 2.0) measured by Nitrogen-13 ammonia can predict all-cause mortality and MACE in the long-term follow-up of patients with chest pain and normal or near-normal coronary arteries (< 30% stenosis). This study supports the notion that there is a subgroup of patients with microvascular dysfunction among those referred to have chest pain and no obstructive CAD.

Our results suggest that the subgroup of patients with low stress MBF and MFR have an increased risk for cardiovascular events. Especially, MFR was a good predictor of all-cause mortality. MFR and stress MBF showed to be good predictors of MACE, even when a large number of patients were lost to follow-up. It is therefore probable that the decrease of MFR and/or stress MBF relates to an early stage of CAD. Interestingly, the presence of a decreased MFR was a better predictor of outcome than stress MBF and or other common risk factors for CAD, including hypertension, dyslipidemia, and typical angina. It is possible that some well-known cardiovascular risk factors did not reach statistical significance in our analysis because of a small sample size and a small number of events.^18^ Another possible explanation is that our population had a low prevalence of hypertension and dyslipidemia. This might suggest that our population represents a group of patients that is more likely to undergo a perfusion assessment due to severe refractory or worsened angina instead of an increased amount of cardiovascular risks. However, no patient with normal MFR died during our follow-up, suggesting a superiority of MFR over stress MBF and other risk factors for the prognosis of adverse cardiovascular events.

The present study corroborates the outcomes of previous reports that indicate that patients with chest pain and no obstructive CAD have increased cardiovascular risk.^4^,^19^ Data from several studies suggest that microvascular dysfunction, which is related to an impaired MFR,^2^ might be associated with that increased cardiovascular risk. In patients with suspected CAD, microvascular dysfunction assessed by PET has shown an additional predictive value, independent of the level of stenosis.^8^,^10^,^20^ Microvascular dysfunction diagnosed by PET has also shown a prognostic value in patients with cardiomyopathy, cardiometabolic diseases, and in patients with ischemic heart failure.^11^,^21^–^23^ Of note, Fukushima et al. and Farhad et al. have reported the short-term prognostic value of myocardial perfusion PET in patients with suspected CAD after excluding patients with PET-driven revascularization.^24^,^25^ Furthermore, Fragasso et al. have described a worse prognosis in patients with chest pain, normal coronary arteries, and diminished myocardial perfusion measured by Single Photon Emission Tomography, during a mean follow-up of 5 years.^19^ Our results are in line with previous reports indicating that microvascular dysfunction, either demonstrated by semi-quantitative or quantitative myocardial perfusion, is a predictor of outcomes. Similar to those studies, our results showed that MFR is a better predictor of outcome when compared to stress MBF. Even though several studies have shown the prognostic value of PET in patients with different degrees of CAD, our study is different from previous. Firstly, our study reports a longer follow-up. Secondly, this study reports a non-invasive quantitative assessment of the microvasculature of patients with normal or near-normal coronary arteries (< 30% stenosis), according to invasive angiography or coronary computed tomography angiography.^2^,^26^

Assessing the cardiovascular risk of patients with chest pain and normal coronary arteries remains a challenge in the clinical setting. On the one hand, our results indicate that patients with preserved microvascular function have an excellent long-term prognosis. On the other hand, our results suggest that an impairment of microvascular function may predict the onset of adverse cardiovascular events. These findings support the clinical value of quantitative myocardial perfusion in the prediction of outcome and encourage an intensification of preventive strategies in this group of patients. The present study also justifies further research that might improve long-term prognosis in patients with both, chest pain, normal or near-normal coronary arteries, and microvascular dysfunction (microvascular angina). Since recent attention has been given to the role of cardiac magnetic resonance as a clinical tool for the assessment of microvascular dysfunction, similar future research could also be conducted to determine its effectiveness as a clinical tool for the assessment of prognosis in this group of patients.^27^–^29^

This study has some limitations. It is a retrospective study, with a small sample size and with few events. The small sample size and few events did not allow us to perform a multivariate analysis.^18^ Even though caution must be applied while extrapolating our results, we believe our sample might be representative of the region since our medical center is a high volume reference for invasive cardiac procedures. Another limitation is that we did not study the role of semi-quantitative analysis of myocardial perfusion images. However, quantitative image analysis might be superior to semi-quantification due to less observer variability. Another limitation was that loss to follow-up was seen in a large percentage of patients for the assessment of MACE. Even though previous simulation studies have reported no important bias in a loss to follow-up of 5-60% of patients,^28^ extra caution was taken using sensitivity analyses that supported the validity of our results.

In conclusion, quantitative myocardial perfusion, measured by Nitrogen-13 ammonia PET, may be a reliable tool to predict all-cause mortality and MACE in patients with chest pain and normal or near-normal coronary arteries even a decade before adverse cardiovascular events occur. Our results indicate that especially MFR can help clinicians to identify those patients who would benefit from a therapy aimed at preventing future cardiovascular events and to relieve symptoms of angina. Future long-term prospective studies are needed in order to better classify and improve microvascular function in this group of patients.

## New Knowledge Gain

In the present study, microvascular angina was related to all-cause mortality and MACE during long-term follow-up. Quantitative myocardial perfusion may adequately predict the incidence of cardiovascular events even a decade before onset in this group of patients.

## Electronic supplementary material

Below is the link to the electronic supplementary material.

Supplementary material 1 (DOCX 26 kb)

Supplementary material 2 (PPTX 317 kb)

## Abbreviations

MBF: Myocardial blood flow
MFR: Myocardial flow reserve
MACE: Mayor adverse cardiac event
CAD: Coronary artery disease
PET: Positron emission tomography
