# Supplementary material for: Long-term prognostic value of quantitative myocardial perfusion in patients with chest pain and normal coronary arteries
Source: J Nucl Cardiol. 2018 Oct 4;26(6):1844–52. doi: 10.1007/s12350-018-1448-8 (PMC6908551; doi:10.1007/s12350-018-1448-8)
Supplement: Supplementary file 1 — Supplementary material 1 (DOCX 26 kb) [file 12350_2018_1448_MOESM1_ESM.docx]

**SUPPLEMENTARY MATERIAL**

**Supplementary Table 1.** Baseline characteristics of patients undergoing a scan in different cameras

|  | All patients n= 79 | ECAT-951/31 PET  n= 50 | ECAT Exact HR+ PET  n= 23 | PET/CT scanner Biograph True Point n= 6 | p- value |
| --- | --- | --- | --- | --- | --- |
| Age (years) | 51 ± 11 | 50 ± 11 | 52 ± 12 | 60 ± 5 | 0.12 |
| Female gender | 59 (74%) | 34 ( 68% ) | 21 ( 91% ) | 4 ( 67% ) | 0.06 |
| Diabetes Mellitus | 3 (4%) | 2 ( 4% ) | 1 ( 4% ) | 0 ( 0% ) | 0.84 |
| Hypertension | 27 (34%) | 14 ( 28 % ) | 9 ( 39 % ) | 4 ( 67 % ) | 0.12 |
| Dyslipidaemia | 22 (28%) | 17 ( 34 % ) | 3 ( 13 % ) | 2 ( 33 % ) | 0.14 |
| Smoker | 12 (18%) | 14 ( 26% ) | 1 ( 4 % ) | 1 ( 13 % ) | 0.07 |
| Body Mass Index | 26 ± 5 | 27 ± 5 | 25 ± 4 | 30 ±10 | 0.19 |
| Typical angina | 43 (54%) | 23 (46%) | 9 (39%) | 4 (67%) | 0.48 |
| Dyspnea | 31 (39%) | 20 (40%) | 7 (30%) | 4 (67%) | 0.27 |
| Rest MBF (ml/gr/min) | 1.0 ± 0.3 | 1.1 ± 0.3 **† *** | 0.8 ± 0.2 **†** | 0.7 ± 0.1 ***** | <0.001 |
| Stress MBF (ml/gr/min) | 2.1 ± 0.6 | 2.1 ± 0.5 | 1.9 ± 0.8 **º** | 2.6 ± 1.0 **º** | 0.05 |
| MPR | 2.3 ± 0.9 | 2.1 ± 0.7 ***** | 2.4 ± 0.9 **º** | 3.5 ± 1.1 *** º** | <0.001 |

**Bonferroni posthoc analysis: †: p<0.05 ; , *: p<0.05; º : p<0.05**

**Supplementary Table 2.** Univariate Cox regressions showing predictors of MACE in patients with complete follow-up

| n = 44 | Hazard Ratio | Lower 95% CI | Upper 95% CI | p-value |
| --- | --- | --- | --- | --- |
| Age | 1.07 | 0.98 | 1.16 | 0.10 |
| Female gender | 1.48 | 0.17 | 12.75 | 0.72 |
| Hypertension | 1.88 | 0.36 | 9.84 | 0.46 |
| Dyslipidemia | 2.22 | 0.44 | 11.17 | 0.33 |
| Smoker | 1.15 | 0.26 | 8.48 | 0.66 |
| Typical angina | 1.57 | 0.31 | 7.81 | 0.58 |
| Dyspnea | 1.59 | 0.32 | 7.96 | 0.57 |
| Increment per unit of stress MBF (ml/gr/min) | 0.11 | 0.02 | 0.83 | 0.03 |
| Increment per unit of MFR | 0.05 | 0.01 | 0.36 | <0.01 |

MBF: myocardial blood flow; MFR: myocardial flow reserve

**Supplementary Table 3.** Univariate Cox regressions showing predictors of MACE after multiple imputation

| n = 79 | Hazard Ratio | Lower 95% CI | Upper 95% CI | p-value |
| --- | --- | --- | --- | --- |
| Age | 1.06 | 0.99 | 1.14 | 0.10 |
| Female gender | 1.24 | 0.21 | 7.30 | 0.81 |
| Hypertension | 1.46 | 0.33 | 6.42 | 0.61 |
| Dyslipidemia | 1.86 | 0.46 | 0.754 | 0.38 |
| Smoker | 2.28 | 0.49 | 10.57 | 0.29 |
| Typical angina | 1.79 | 0.44 | 7.17 | 0.41 |
| Dyspnea | 1.61 | 0.41 | 6.10 | 0.51 |
| Increment per unit of stress MBF (ml/gr/min) | 0.16 | 0.02 | 0.93 | 0.03 |
| Increment per unit of MFR | 0.06 | 0.005 | 0.69 | 0.02 |

MBF: myocardial blood flow; MFR: myocardial flow reserve

Outcome and time to event were imputed 35 times using the following covariates: age, gender, hypertension, dyslipidemia, history of smoking, typical angina, dyspnea, stress myocardial blood flow, myocardial flow reserve, and complete outcome and time to event information.
