# Supplementary material for: Long-term prognostic value of quantitative myocardial perfusion in patients with chest pain and normal coronary arteries
Source: J Nucl Cardiol. 2018 Oct 4;26(6):1844–52. doi: 10.1007/s12350-018-1448-8 (PMC6908551; doi:10.1007/s12350-018-1448-8)
Supplement: Supplementary file 2 — Supplementary material 2 (PPTX 317 kb) [file 12350_2018_1448_MOESM2_ESM.pptx]

## Slide 1
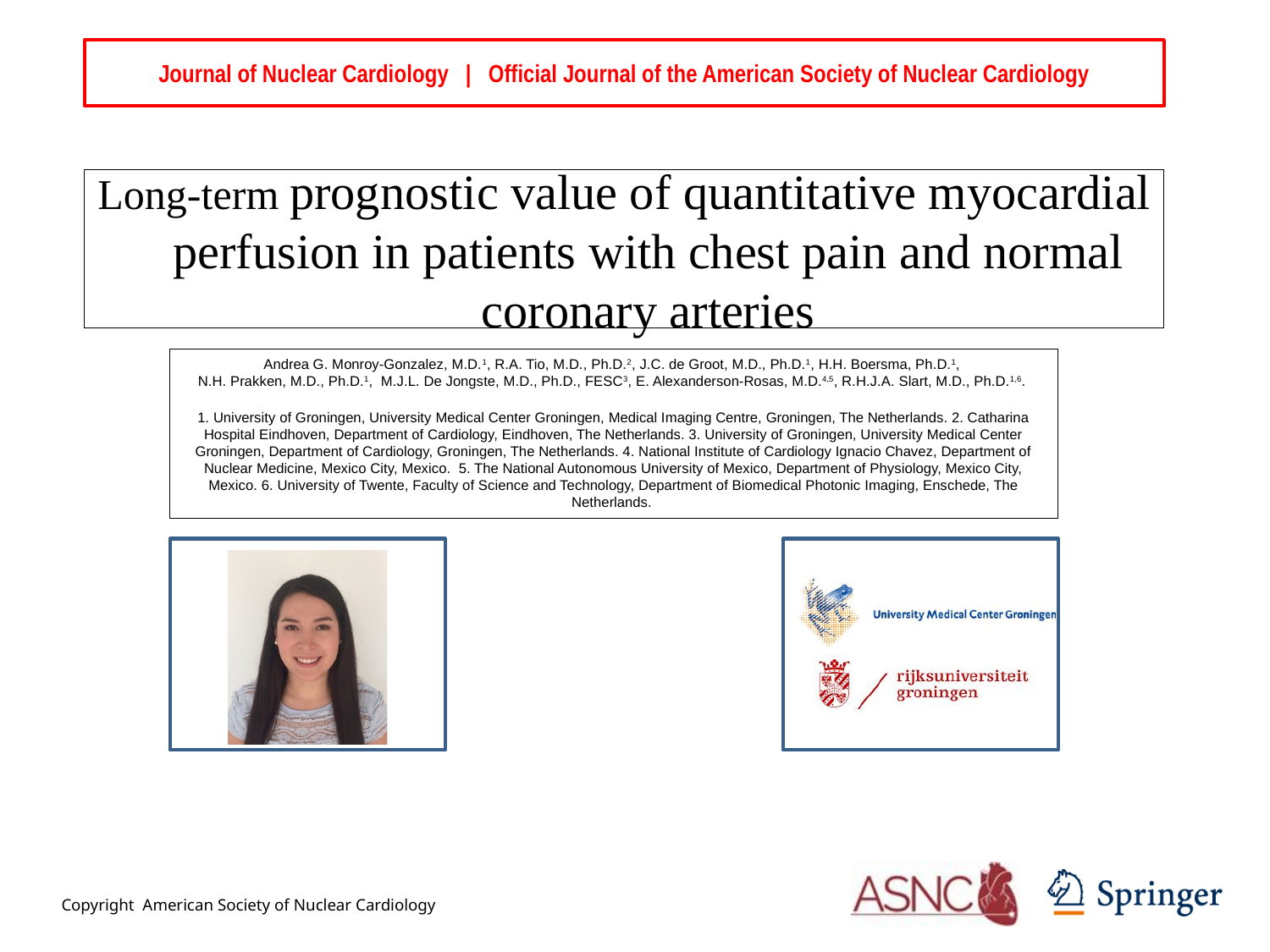

Journal of Nuclear Cardiology | Official Journal of the American Society of Nuclear Cardiology
# Long-term prognostic value of quantitative myocardial perfusion in patients with chest pain and normal coronary arteries
Andrea G. Monroy-Gonzalez, M.D.1, R.A. Tio, M.D., Ph.D.2, J.C. de Groot, M.D., Ph.D.1, H.H. Boersma, Ph.D.1,
N.H. Prakken, M.D., Ph.D.1, M.J.L. De Jongste, M.D., Ph.D., FESC3, E. Alexanderson-Rosas, M.D.4,5, R.H.J.A. Slart, M.D., Ph.D.1,6.
1. University of Groningen, University Medical Center Groningen, Medical Imaging Centre, Groningen, The Netherlands. 2. Catharina Hospital Eindhoven, Department of Cardiology, Eindhoven, The Netherlands. 3. University of Groningen, University Medical Center Groningen, Department of Cardiology, Groningen, The Netherlands. 4. National Institute of Cardiology Ignacio Chavez, Department of Nuclear Medicine, Mexico City, Mexico. 5. The National Autonomous University of Mexico, Department of Physiology, Mexico City, Mexico. 6. University of Twente, Faculty of Science and Technology, Department of Biomedical Photonic Imaging, Enschede, The Netherlands.
Copyright American Society of Nuclear Cardiology

## Slide 2
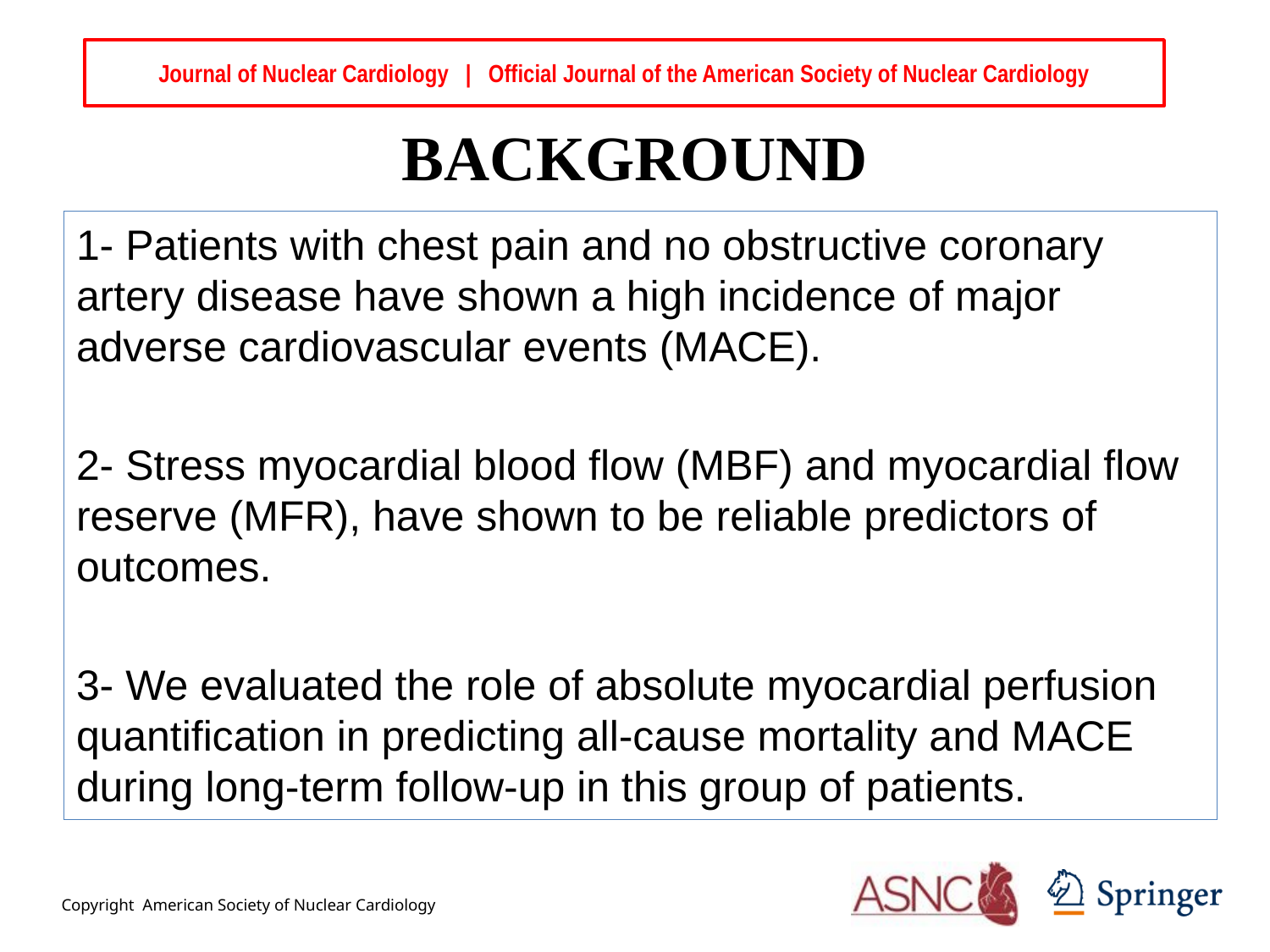

Journal of Nuclear Cardiology | Official Journal of the American Society of Nuclear Cardiology
# BACKGROUND
1- Patients with chest pain and no obstructive coronary artery disease have shown a high incidence of major adverse cardiovascular events (MACE).
2- Stress myocardial blood flow (MBF) and myocardial flow reserve (MFR), have shown to be reliable predictors of outcomes.
3- We evaluated the role of absolute myocardial perfusion quantification in predicting all-cause mortality and MACE during long-term follow-up in this group of patients.
Copyright American Society of Nuclear Cardiology

## Slide 3
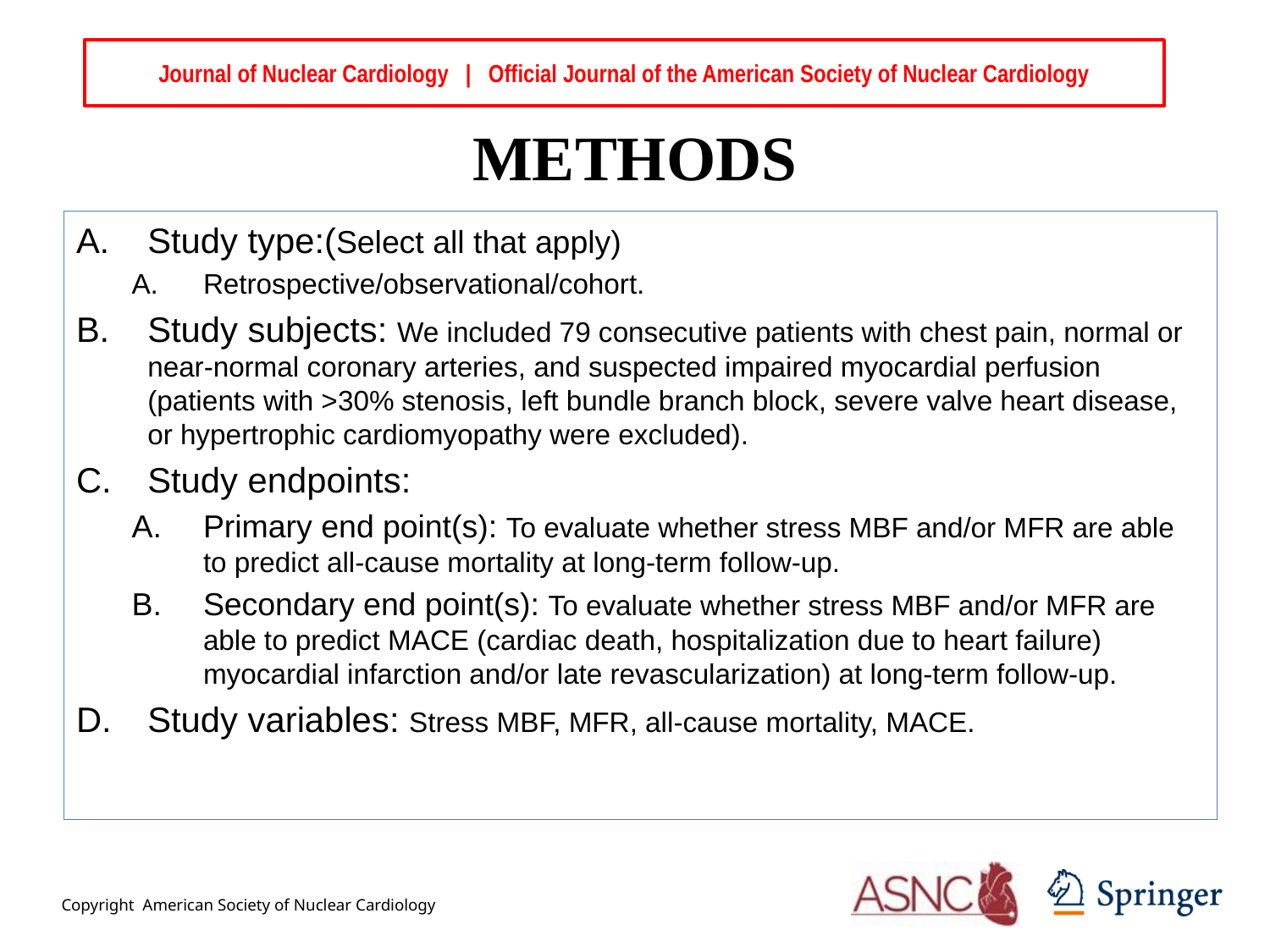

Journal of Nuclear Cardiology | Official Journal of the American Society of Nuclear Cardiology
# METHODS
Study type:(Select all that apply)
Retrospective/observational/cohort.
Study subjects: We included 79 consecutive patients with chest pain, normal or near-normal coronary arteries, and suspected impaired myocardial perfusion (patients with >30% stenosis, left bundle branch block, severe valve heart disease, or hypertrophic cardiomyopathy were excluded).
Study endpoints:
Primary end point(s): To evaluate whether stress MBF and/or MFR are able to predict all-cause mortality at long-term follow-up.
Secondary end point(s): To evaluate whether stress MBF and/or MFR are able to predict MACE (cardiac death, hospitalization due to heart failure) myocardial infarction and/or late revascularization) at long-term follow-up.
Study variables: Stress MBF, MFR, all-cause mortality, MACE.
Copyright American Society of Nuclear Cardiology

## Slide 4
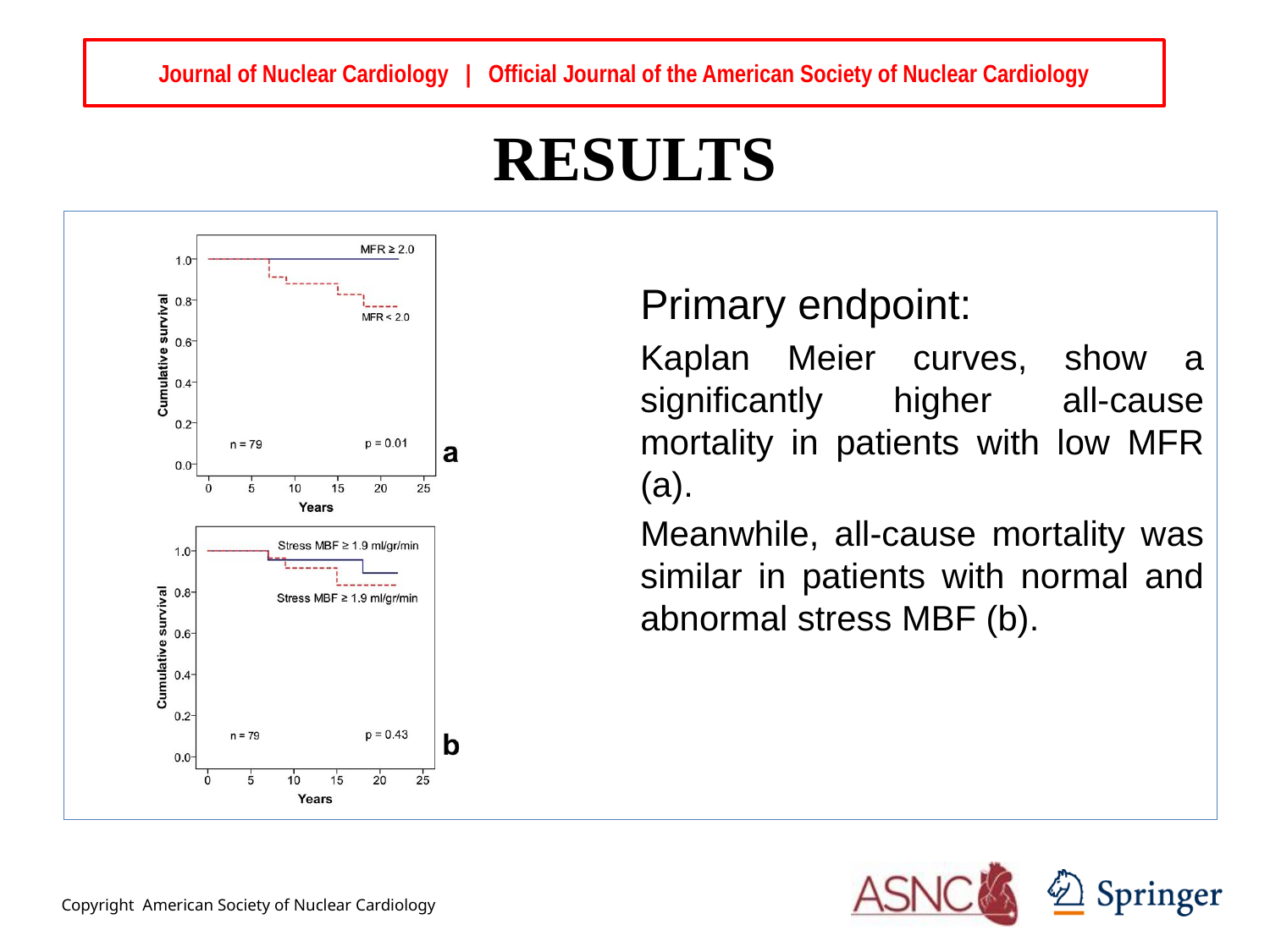

Journal of Nuclear Cardiology | Official Journal of the American Society of Nuclear Cardiology
# RESULTS
Primary endpoint:
Kaplan Meier curves, show a significantly higher all-cause mortality in patients with low MFR (a).
Meanwhile, all-cause mortality was similar in patients with normal and abnormal stress MBF (b).
Copyright American Society of Nuclear Cardiology

## Slide 5
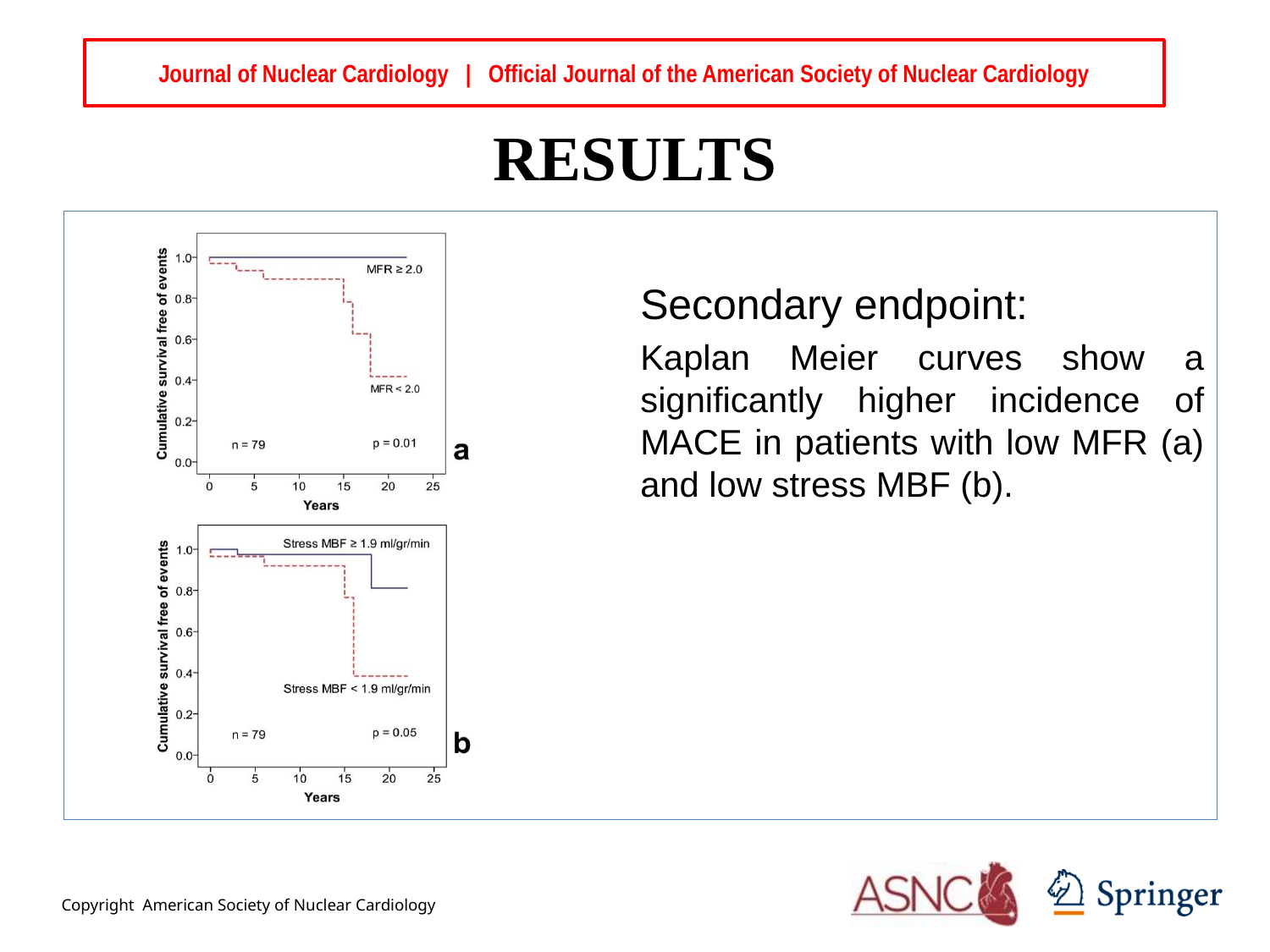

Journal of Nuclear Cardiology | Official Journal of the American Society of Nuclear Cardiology
# RESULTS
Secondary endpoint:
Kaplan Meier curves show a significantly higher incidence of MACE in patients with low MFR (a) and low stress MBF (b).
Copyright American Society of Nuclear Cardiology

## Slide 6
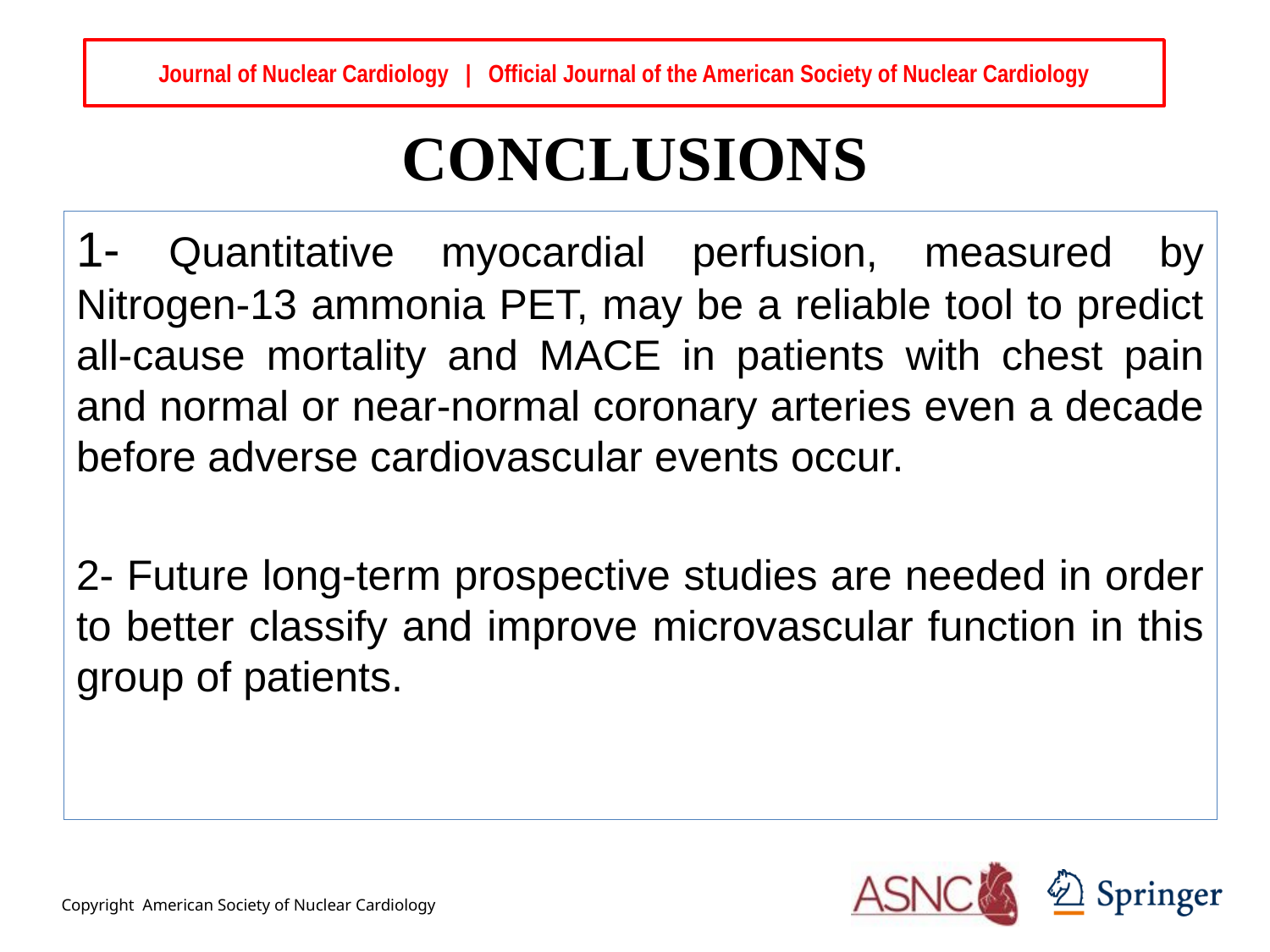

Journal of Nuclear Cardiology | Official Journal of the American Society of Nuclear Cardiology
# CONCLUSIONS
1- Quantitative myocardial perfusion, measured by Nitrogen-13 ammonia PET, may be a reliable tool to predict all-cause mortality and MACE in patients with chest pain and normal or near-normal coronary arteries even a decade before adverse cardiovascular events occur.
2- Future long-term prospective studies are needed in order to better classify and improve microvascular function in this group of patients.
Copyright American Society of Nuclear Cardiology
